# Differential regulation of MYC expression by *PKHD1/Pkhd1* in human and mouse kidneys: phenotypic implications for recessive polycystic kidney disease

**DOI:** 10.3389/fcell.2023.1270980

**Published:** 2023-11-17

**Authors:** Naoe Harafuji, Chaozhe Yang, Maoqing Wu, Girija Thiruvengadam, Heather Gordish-Dressman, R. Griffin Thompson, P. Darwin Bell, Avi Z. Rosenberg, Claudia Dafinger, Max C. Liebau, Zsuzsanna Bebok, Ljubica Caldovic, Lisa M. Guay-Woodford

**Affiliations:** ^1^ Center for Translational Research, Children’s National Hospital, Washington, DC, United States; ^2^ Heersink School of Medicine, The University of Alabama at Birmingham, Birmingham, AL, United States; ^3^ Department of Pathology, Johns Hopkins University, Baltimore, MD, United States; ^4^ Department of Pediatrics and Center for Molecular Medicine, Medical Faculty and University Hospital Cologne, University of Cologne, Cologne, Germany; ^5^ Department of Pediatrics, Center for Family Health, Center for Rare Diseases and Center for Molecular Medicine, Medical Faculty and University Hospital Cologne, University of Cologne, Cologne, Germany; ^6^ Center for Genetic Medicine Research, Children’s National Hospital, Washington, DC, United States; ^7^ Department of Genomics and Precision Medicine, School of Medical and Health Sciences, The George Washington University, Washington, DC, United States

**Keywords:** ARPKD, MYC, FPC, cystin, PKHD1, Cys1

## Abstract

Autosomal recessive polycystic kidney disease (ARPKD; MIM#263200) is a severe, hereditary, hepato-renal fibrocystic disorder that leads to early childhood morbidity and mortality. Typical forms of ARPKD are caused by pathogenic variants in the *PKHD1* gene, which encodes the fibrocystin/polyductin (FPC) protein. MYC overexpression has been proposed as a driver of renal cystogenesis, but little is known about MYC expression in recessive PKD. In the current study, we provide the first evidence that MYC is overexpressed in kidneys from ARPKD patients and confirm that MYC is upregulated in cystic kidneys from *cpk* mutant mice. In contrast, renal MYC expression levels were not altered in several *Pkhd1* mutant mice that lack a significant cystic kidney phenotype. We leveraged previous observations that the carboxy-terminus of mouse FPC (FPC-CTD) is proteolytically cleaved through Notch-like processing, translocates to the nucleus, and binds to double stranded DNA, to examine whether the FPC-CTD plays a role in regulating *MYC/Myc* transcription. Using immunofluorescence, reporter gene assays, and ChIP, we demonstrate that both human and mouse FPC-CTD can localize to the nucleus, bind to the *MYC/Myc* P1 promoter, and activate *MYC/Myc* expression. Interestingly, we observed species-specific differences in FPC-CTD intracellular trafficking. Furthermore, our informatic analyses revealed limited sequence identity of FPC-CTD across vertebrate phyla and database queries identified temporal differences in *PKHD1*/*Pkhd1* and *CYS1*/*Cys1* expression patterns in mouse and human kidneys. Given that cystin, the *Cys1* gene product, is a negative regulator of *Myc* transcription, these temporal differences in gene expression could contribute to the relative renoprotection from cystogenesis in *Pkhd1*-deficient mice. Taken together, our findings provide new mechanistic insights into differential mFPC-CTD and hFPC-CTD regulation of MYC expression in renal epithelial cells, which may illuminate the basis for the phenotypic disparities between human patients with *PKHD1* pathogenic variants and *Pkhd1*-mutant mice.

## 1 Introduction

The *MYC* proto-oncogene, encoding the MYC transcription factor, was first identified in patients with Burkitt’s lymphoma (Taub et al., 1982). MYC contributes to the regulation of multiple cellular signaling pathways involved in cell proliferation (Gearhart et al., 2007). Aberrant MYC expression induces malignant transformation of several tumor types (Dang, 2012; Gabay et al., 2014). In addition, MYC increases the expression of inflammatory and fibrotic factors, which may significantly contribute to the pathogenesis of cystic kidney diseases (Nevzorova et al., 2013; Karihaloo, 2015; Shen et al., 2017). MYC overexpression in renal epithelia has been reported in several mouse ADPKD models as well as in *cpk* mice (Cowley et al., 1991; Burtey et al., 2008; Kurbegovic and Trudel, 2013; Wu et al., 2013). Elevated MYC appears to be a signature of renal cystic disease and may define a causative pathway (Trudel, 2015; Parrot et al., 2019). However, the role of MYC activation in the initiation and progression of autosomal recessive polycystic kidney disease (ARPKD; MIM#263200) remains incompletely understood.

ARPKD is a hereditary hepato-renal fibrocystic disorder with an estimated incidence of 1 in 26,500 live births (Guay-Woodford et al., 2014; Alzarka et al., 2017). Pathogenic variants in the polycystic kidney and hepatic disease 1 (*PKHD1*) gene, located on chromosome 6p21.1, cause all typical forms of human ARPKD. The longest *PKHD1* (MIM#606702) open reading frame (ORF) contains 67 exons, which encode a 4,074 amino acid protein called fibrocystin/polyductin (FPC) (Onuchic et al., 2002; Ward et al., 2002). Full length FPC is a single transmembrane (TM) domain protein predicted to have several immunoglobulin-like IPT/TIG conserved domains, two G8 domains, and multiple parallel beta-helix 1 (PbH1) repeats in a long extracellular segment (3,858 amino acids), and a short (192 amino acids) cytoplasmic C-terminal domain (CTD) (Supplementary Figure S5A) (Sharp et al., 2005; Wang et al., 2007). The *PKHD1* mRNA is primarily expressed in the kidney, liver, lung, and pancreas (Onuchic et al., 2002; Ward et al., 2002; Xiong et al., 2002). In adult and fetal human tissues, FPC is expressed in renal collecting ducts, thick ascending limbs of loops of Henle, bile ducts, pancreatic ducts, epididymis, and testis (Ward et al., 2003; Menezes et al., 2004). Pathogenic sequence variants in the *PKHD1* gene account for more than 80% of human ARPKD cases (Bergmann, 2017). Less than 1% of ARPKD patients have pathogenic sequence variants either in *DZIP1L* or *CYS1* genes (Lu et al., 2017; Yang et al., 2021), and the molecular cause of ARPKD in remaining patients remains to be determined (Bergmann, 2017).

*Pkhd1* is the mouse ortholog of *PKHD1*. The longest ORF of mouse *Pkhd1* also contains 67 exons and encodes a 4,059 amino acid protein. The mouse and human FPC sequences are 73% identical overall but the CTD share only 55% identity (Nagasawa et al., 2002). Mouse FPC is also a single TM domain protein with 3,872 amino acid N-terminal segment and a short (187 amino acids) cytoplasmic CTD. Mouse FPC has the same numbers of conserved IPT/TIG and G8 domains as human FPC (Nagasawa et al., 2002; Bergmann, 2017). The intracellular domain of mouse FPC (mFPC-CTD), contains an 18-residue long ciliary targeting signal (CTS) that facilities delivery to the primary cilium (Follit et al., 2010). The mFPC-CTD, encoded by exons 65-67, undergoes Notch-like processing followed by regulated membrane-release and translocation to the nucleus (Kaimori et al., 2007), which is facilitated by the 25-residue long nuclear localization signal (NLS) (Hiesberger et al., 2006). Single particle electron microscopy analysis revealed that FPC-CTD forms a ring-like protein complex that binds to double stranded DNA, suggesting a role in gene expression regulation (Cameron Varano et al., 2017). Yet, the function of FPC-CTD in the nucleus remains poorly understood. Numerous rodent models of ARPKD with mutations and multiple exon deletions in *Pkhd1* have been generated. However, these models express minimal or no renal disease (Katsuyama et al., 2000; Ward et al., 2002; Masyuk et al., 2004; Moser et al., 2005; Garcia-Gonzalez et al., 2007; Woollard et al., 2007; Gallagher et al., 2008; Kim et al., 2008; Williams et al., 2008; Hu et al., 2011; Outeda et al., 2017; Ishimoto et al., 2023).

The most widely studied mouse model of ARPKD, the *cpk* mouse carries a spontaneous insertion/deletion (indel) mutation in the *Cys1* gene, encoding the cystin protein (Hou et al., 2002; Guay-Woodford, 2003; Nagao et al., 2012). The renal phenotype of *cpk* mice closely resembles human ARPKD. Mouse cystin, the product of the *Cys1* gene, is a 145-amino acid, cilia-associated protein that is mainly expressed in mouse kidney and liver ductal epithelium as early as embryonic day 14.5 (Tao et al., 2009). Mouse cystin contains a predicted N-myristylation motif (MGSGSSR) and a NLS located in the first 27 amino acids at the N-terminus. Amino acids 28-35 of mouse cystin contain a cilium-targeting motif (AxEGG) that is required for cystin trafficking to the primary cilium (Tao et al., 2009). Our previous demonstration that cystin suppresses *Myc* transcription by binding to necdin, an activator of the *Myc* P1 promoter, links renal cystogenesis in *cpk* mice to *Myc* activation and enhanced MYC levels (Wu et al., 2013; Yang et al., 2021).

Human *CYS1* encodes Cystin-1, a 158-amino acid protein (Fliegauf et al., 2003). Sequence comparison of human and mouse orthologs Cystin-1 and cystin shows 57% identity and 64% similarity (Fliegauf et al., 2003). Initial analysis of *CYS1* expression in adult human tissues revealed high *CYS1* mRNA abundance in the kidney and pancreas (Fliegauf et al., 2003). Subsequent RNA-seq analysis of human tissues consistently revealed high *CYS1* mRNA levels in the kidney and lower expression in several other tissues including ovary, gall bladder, endometrium, pancreas, and lung (Fagerberg et al., 2014). The function of Cystin-1 is not understood, although we have reported the first genetic defect in human *CYS1* that causes the renal ARPKD phenotype (Yang et al., 2021).

In the current study, we employed immunofluorescence imaging as well as bioinformatic, molecular, and biochemical analyses to comparatively evaluate the roles of human and mouse FPC-CTDs in the regulation of MYC expression in human and mouse renal epithelia.

## 2 Materials and methods

### 2.1 Human samples

All human studies were approved by the Institutional Review Board at the Children’s National Hospital or the University Hospital of Cologne. Human kidney samples were obtained from the NIDDK-funded UAB Childhood Cystic Kidney Disease Center Translational Resource at the University of Alabama at Birmingham and the University Hospital of Cologne. Kidney samples were obtained from patients ranging from 26 weeks of gestation age to 3 years of age (Supplementary Table S1).

### 2.2 Animal study approval

All mouse experiments were approved by the Institutional Animal Care and Use Committees at Children’s National Research Institute, and experiments were carried out in accordance with relevant guidelines and regulations. Mouse colonies were maintained in the animal facility at Children’s National Research Institute. All mouse kidneys were harvested from 14-day-old, 10- and 12-month-old mutants and age-matched wild-type (WT) littermates. Genetic background information for all mouse lines used in this study is shown in Supplementary Table S2.

### 2.3 Antibodies

All antibodies used for this study are listed in the Supplementary Table S3, unless specified in the text.

### 2.4 Immunohistochemistry (IHC)

Immunohistochemical staining for MYC was performed on formalin-fixed, paraffin embedded tissues using heat induced epitope retrieval solution (BOND Epitope Retrieval Solution 2, Leica Biosystems, Cat. No. AR9640) and an automated stainer (Bond-Max, Leica Biosystems). Tissues were incubated with anti-MYC antibody (Recombinant Anti-c-Myc antibody [Y69] - ChIP Grade, Abcam) at 1:25 dilution for 120 min.

### 2.5 Immunoblotting

Cultured cells and kidney tissues were collected, homogenized, and processed for immunoblotting as previously described (Wu et al., 2013; Dafinger et al., 2020). Immuno-reactive protein bands were visualized using SuperSignal West Femto Maximum Sensitivity Substrate (Thermo Fisher Scientific, Cat. No. 34095) and images were obtained with ChemiDoc Imaging System (Bio-Rad laboratory). Densitometry was performed using Image Lab software (Bio-Rad laboratory, Version 6.0).

### 2.6 RNA extraction and qRT-PCR

Kidney tissue samples from 14-day-old male mice were snap frozen, transferred to a gentleMACS M tubes (Miltenyi Biotec, Cat. No. 130-093-236, RRID:SCR_020269) in Buffer RLT plus 2-Mercaptoethanol (as per RNeasy Mini Kit instructions, Qiagen, Cat. No. 74104), and homogenized using a gentleMACS Dissociator (Miltenyi Biotec) per the manufacturer’s program RNA-02. Homogenized samples were transferred to microcentrifuge tubes for total RNA extraction using the RNeasy Mini Kit according to the manufacturer’s instructions. Total RNA from both 5-day-postconfluent mIMCD-3 cells stably expressing FPC-CTD and hTERT-immortalized human renal epithelial cells (hTERT-HRE) transiently transfected with FPC-CTD were isolated using the RNeasy Mini Kit (Qiagen) according to the manufacturer’s instructions. Isolated total RNA was treated with RQ1 RNase-Free DNase (Promega, Cat. No. M6101), and then repurified using the RNeasy Mini kit. RNA samples were reverse-transcribed using SuperScript III First-Strand Synthesis SuperMix (Thermo Fisher Scientific, Cat. No. 18080400) and oligo dT primers as described in the manufacturer’s instructions.

Quantitative RT-PCR was performed on a QuantStudio 7 Flex Real-Time PCR System (Thermo Fisher Scientific) using the default program. The PCR was performed with cDNA templates using Power SYBR Green PCR Master Mix (Thermo Fisher Scientific, Cat. No. 4368706) and mouse primers specific for sequences of *Myc* (forward: 5′- GCC CCC AAG GTA GTG ATC CT -3’; reverse: 5′- GTG CTC GTC TGC TTG AAT GG -3′). Peptidylprolyl isomerase A (*PPIA*) was used for normalization (forward: 5′- AGC ACT GGA GAG AAA GGA TT -3’; reverse: 5′- ATT ATG GCG TGT AAA GTC ACC A-3′) (Arensdorf and Rutkowski, 2013). Overexpression of FPC-CTD in cell lines was confirmed with *Pkhd1* exon 66–67 specific primers (forward: 5′-CCA GAA GAC ATA TCT GAA TCC CAG GC-3’; reverse: 5′-AGC AAG AGA TCC TGG AAC ACA GGT-3′). Results were analyzed using QuantStudio Real-Time PCR Software and the ΔΔCt method (Livak and Schmittgen, 2001).

### 2.7 Conservation analysis of vertebrate *Pkhd1* gene products (fibrocystin/polyductin (FPC)) using bioinformatics tools

Protein sequences of FPC were collected from NCBI protein database (https://www.ncbi.nlm.nih.gov/protein/) using an advanced search with gene name, *Pkhd1* and taxonomic groups Mammalia, Aves, Reptilia, Amphibia, Caecilians, and Fish. This resulted in 102 FPC sequences from mammals, birds, reptiles, amphibians, and fish (Supplementary Table S4). FPC sequence from each species was verified by protein alignment with human FPC; the protein sequences that were significantly shorter than human FPC sequence were removed. WebLogo 3 (Crooks et al., 2004) was used to visualize FPC sequence alignment (Supplementary Figure S1) that was generated with Clustal Omega (Sievers et al., 2011). The conservation scores of FPC amino acids were extracted from the WebLogo 3 raw data (Supplementary Table S5). Conserved domains in FPC were mapped by Conserved Domain Database (CDD) (Lu et al., 2020).

Prediction of nuclear localization signals (NLSs) were performed with the human and mouse FPC-CTD construct sequences using SeqNLS (Lin et al., 2012) with 0.86 as the cut-off score.

### 2.8 Plasmid construction

*pcDNA5/FRT/TO (pcDNA5)* was obtained from Thermo Fisher Scientific (Cat. No. V652020).

*pcDNA5/FRT/TO-mPkhd1-CTD-V5 (pcDNA5-mFPC-CTD)*: the cytoplasmic tail of mouse FPC (Follit et al., 2010) expression construct was generated from pcDNA5/FRT/TO-mPkhd1 (full length) -V5 (gift from Dr. Feng Qian) with site-directed mutagenesis (SDM) using 5′- GCT AAC TGG ACA TGA TGC TTT GCT GCT GGT TTA AGA AAA GC -3′ and 5′- GCT TTT CTT AAA CCA GCA GCA AAG CAT CAT GTC CAG TTA GC -3′ primer set.

*pcDNA5/FRT/TO-mPkhd1-CTD* ^*delCTS*^ *-V5* (*pcDNA5-mFPC-CTD* ^*delCTS*^): the ciliary targeting sequence (Follit et al., 2010) deleted mFPC-CTD expression construct was made from mFPC-CTD by SDM with 5′- GCT AAC TGG ACA TGA TGC TTG ACA TAT CTG AAT CCC AGG CT -3′ and 5′- AGC CTG GGA TTC AGA TAT GTC AAG CAT CAT GTC CAG TTA GC -3′ primer set.

*pcDNA5/FRT/TO-hPKHD1-CTD-V5 (pcDNA-hFPC-CTD)*: the expression construct containing the hFPC-CTD, comparable to mFPC-CTD (Follit et al., 2010), and fused to V5-tag was made by LifeSct LLC. The hFPC-CTD coding sequence was cloned between *KpnI* and *NotI* sites in pcDNA5/FRT/TO.

*pcDNA5/FRT/TO-hPKHD1-CTD* ^*delCTS*^ *-V5* (*pcDNA-hFPC-CTD* ^*delCTS*^): the ciliary targeting sequence deleted hFPC-CTD expression construct was made from hFPC-CTD by SDM using 5′- CCG TGG ACA GAA TGA CTG CCG AGA TTC CTG AAT CCC AGA C -3′ and 5′- GTC TGG GAT TCA GGA ATC TCG GCA GTC ATT CTG TCC ACG G -3′ primer set.

*pGL4.22 [luc2CP/Puro] vector* was purchased from Promega (Cat. No. E6771).

*pRL-TK* *vector* was purchased from promega (Cat. No. E2241).

*pGL4.22-mouse Myc P1 (pGL4.22-mMyc P1)*: the mouse *Myc* P1 promoter (chr8:127735983-127736125, GRCm38/mm10 mouse genome assembly) construct was described previously (Wu et al., 2013).

*pGL4.22-human MYC P1 (pGL4.22-hMYC P1)*: the human *MYC* P1 promoter (chr15:61985298-61985433, GRCh38/hg38 human genome assembly), which is comparable to the mouse *Myc* P1 promoter, was amplified by PCR from HEK293 genomic DNA and cloned into pGL4.22 vector at *XhoI* and *HindIII* sites using 5′- CCG CTC GAG GAG GGC GTG GGG GAA AAG A-3′ and 5′- CCC AAG CTT AGC CAG GGA CGG CCG G -3′ primer set. Sequence alignment of human and mouse *MYC/Myc* P1 promoter shown in Supplementary Figure S2 was created by Clustal Omega (Sievers et al., 2011).

### 2.9 Cell culture and generation of stable cell lines expressing FPC-CTDs

*Mouse TERT immortalized cortical collecting duct (mTERT-CCD) cells* (Steele et al., 2010) were cultured in TERT culture medium [DMEM/F-12 medium (Thermo Fisher Scientific, Cat. No. 11330057) containing 5% heat-inactivated fetal bovine serum (FBS) (Atlanta Biologicals, Cat. No. S11050H), 1% penicillin/streptomycin (Thermo Fisher Scientific, Cat. No. 15140163), 1x Insulin-Transferrin-Selenium solution (Thermo Fisher scientific, Cat. No. 41400045), 0.2 μg/mL dexamethasone (Sigma-Aldrich, Cat. No. D8893) and 10 nM 3,3′,5-Triiodo-L-thyronine sodium salt (Sigma-Aldrich, Cat. No. T6397)] at 37°C in 5% CO_2_.

*Mouse inner medullary collecting duct (mIMCD)-3 cells* were purchased from American Type Culture Collection (ATCC, Cat. No. CRL-2123) and cultured in complete growth medium (CGM) [DMEM/F-12 medium (Thermo Fisher Scientific, Cat. No. 11330057) containing 10% heat-inactivated fetal bovine serum (Atlanta Biologicals, Cat. No. S11050H) and 1% penicillin/streptomycin (Thermo Fisher Scientific, Cat. No. 15140163)] at 37°C in 5% CO_2_.

*mIMCD-3 mFPC-CTD stable cell lines* were generated by transfection of either pcDNA5-mFPC-CTD or pcDNA5 into mIMCD-3 cells with Lipofectamine2000 transfection reagent (Thermo Fisher Scientific, Cat. No. 11668019). At 48 hrs post-transfection, cells with spontaneously integrated plasmids were selected with hygromycin (1 mg/mL) (Thermo Fisher Scientific, Cat. No. 10687010) for 1 week. The mFPC-CTD stably overexpressing cells and the control empty vector cells were then maintained in CGM with hygromycin (200 μg/mL).

### 2.10 Generating human TERT-immortalized renal epithelial cell line (hTERT-HRE)

Human kidney sections were minced and immediately placed in 1% collagenase type I (Sigma, Cat. No. C0130-1G) in DMEM/F12 (Thermo Fisher, Cat No. 11330032) and incubated on a rotator for 30 min at room temperature (RT). Renal epithelial cells and tubule fragments were then transferred to a conical tube containing DMEM/F12 and centrifuged at 750 *g* for 10 min. The supernatant was removed, and the tissue was resuspended in a complete medium, containing 0.2 mg/mL dexamethasone (Sigma, Cat. No. D8893-1 MG), 5% heat inactivated FBS (Hyclone, Cat. No. SH30396-03), 2 mM glutamate (Thermo Fisher, Cat. No. 25030081), 1x insulin-transferrin-sodium selenite (ITS) (Thermo Fisher, Cat. No. 1400045), 100 U/mL penicillin/streptomycin (Thermo Fisher, Cat. No. 15140122), and 10 nM triiodothyronine (Sigma, Cat. no. T6397-100 MG) in DMEM/F12. No antibiotics were added to the complete medium in preparation for the transduction with the hTERT lentiviral expression construct. Cells were maintained in a 37°C humidified incubator with 5% CO_2_.

Lenti-hTERT-Neo Virus (Cat. No. LV622), Lenti-p53 siRNA Virus (Cat. No. G219), and Polybrene (Cat. No. G062) were purchased from Applied Biological Materials (Richmond, BC, Canada). One day prior to transfection, primary human renal epithelial cells were plated at 20%–30% confluency in a 6 well plate. The next day, the complete medium was replaced with 1 mL of transfection medium, which contained 6 μg/mL of polybrene in the complete medium. Then, 1.54 × 10^8^ transducing units (TU)/ml of Lenti-hTERT-Neo Virus and 1 × 10^6^ TU/mL of Lenti-p53 siRNA Virus were added to the transfection medium at a multiplicity of infection (MOI) of 7. The plate was centrifuged at 200 *g* for 30 min and then placed back in the incubator at 37°C. After 24 h culture at 37°C, 1 mL of the complete medium was added to each 6 well and cultured for an additional 24 h. Cells immortalized with the hTERT gene then were selected at 48 h post transduction, using 800 μg/mL G418 in the complete medium.

Characterization of the hTERT-HRE cells was performed using immunoblotting of E-cadherin as an epithelial marker, ZO-1 as a tight junction marker, α-ENaC as a renal epithelial cell marker, AQP1 as a renal tubule cell marker, and Keratin 17/19 as an epithelial cell marker (Supplementary Figure S3A). In addition, RT-PCR was performed for *PKHD1* as a renal epithelial marker (Supplementary Figure S3B).

### 2.11 Immunocytochemistry

For MYC immunofluorescence staining, cells stably overexpressing mFPC-CTD were seeded onto coverslips in 6-well plates and cultured until confluent. For FPC-CTD localization assay, mTERT-CCD or hTERT-HRE cells were transiently transfected with pcDNA-mFPC-CTD and pcDNA-mFPC-CTDd^elCTS^, or pcDNA5-hFPC-CTD and pcDNA5-hFPC-CTD^delCTS^, respectively. Forty-8 hrs. after transfection, cells were washed with PBS and fixed with 4% paraformaldehyde (PFA) for 10 min at RT and then permeabilized with 0.5% Triton X-100 in PBS for 5 min, followed by three washes with PBS before blocking with 1% BSA for 30 min. The cells were incubated with primary antibodies (anti-V5 or anti-Myc) overnight at 4°C followed by incubation with secondary antibody (Alexa Fluor 488 at 1:400 dilution) for 1 h at RT. The cells were then washed three times with PBS and mounted with ProLong Gold + DAPI (Life Technologies, Cat. No. P36935). Fluorescently labeled cells were analyzed on an Olympus FV1000 scanning laser confocal microscope configured with both an Argon Laser (5 mW, 488 nm), and a Green HeNe (10 mW, 543 nm) laser. Images were analyzed using Olympus FV10-ASW 3.0 Viewer software.

### 2.12 Reporter gene assay

Cells were seeded in 24-well plate, grown to ∼90% confluence, and then transfected with pGL4.22-*mMyc* P1 or pGL4.22*-hMYC* P1): (0.3 µg/well) and pcDNA5-mFPC-CTD (0.6 μg or 1.2 μg/well) or pcDNA5-hFPC-CTD (0.6 µg/well) using Lipofectamine 2000. The differences in transfection efficiency were normalized by co-transfecting with 15 ng/well pRL plasmid that expressed *Renilla* luciferase (Promega) and adjusting the total amount of plasmid DNA to 1.5 ug/well by adding pcDNA5. The transfected cells were incubated for 48 h, lysed in 100 µL/well passive lysis buffer (Promega) and shaken for 20 min at RT. Firefly and *Renilla* luciferase activities were measured with Dual-Luciferase Reporter Assay System (Promega, Cat. No. E1910). The luminometer (FLUOstar OPTIMA, BMG LABTECH) was programmed using OPTIMA software to perform a 0 s delay, followed by a 5-s measurement period for each reporter assay. The 20 μL cell lysate was transferred into 96-well plate (Costar, Cat. No. 3912; white flat bottom), followed by the addition of 100 µL Luciferase Assay Reagent II and luminescence reading. After measurement of firefly luciferase activity, 100 µL of Stop&Glo reagent was added and quickly put back for reading of the *Renilla* luciferase activity. Data were collected from three independent transfections and processed using GraphPad Prism version 9.1.2 for Windows, GraphPad Software, San Diego, California United States of America, www.graphpad.com.

### 2.13 Chromatin immunoprecipitation (ChIP) assay

To determine the binding of FPC-CTD to the *Myc* P1 promoter, experiments were performed using Magna ChIP A/G Chromatin Immunoprecipitation Kit (MilliporeSigma, Cat. No. 17-10085), according to the manufacturer instructions and our previously published protocol (Wu et al., 2013). Because ChIP-grade anti-FPC-CTD antibodies were not available, we generated mIMCD-3 cells stably overexpressing mFPC-CTD-V5 and control cells stably transfected with empty vector and used ChIP-grade anti-V5 antibody (Abcam, Cat. No. ab15828) for immunoprecipitation. Cells were grown to 80%–90% confluence prior to experiments and processed according to the Magna ChIP A/G protocol. Following immunoprecipitation with anti-V5 antibody that recognized mFPC-CTD-V5 bound to the chromatin and subsequent protease digestion, we amplified *Myc* P1 using PCR primers specific to the full-length *Myc* P1 (forward primer: 5′- CGC TCG AGG AGA GAG GTG GGG AAG GGA GAA AG -3’; reverse primer: 5′- CCC AAG CTT AGT GAG GCG AGT CGG ACC CGG CA -3′) using the following PCR program: 94°C 3 min; 94°C 20 s, 62°C 20 s, 72°C 15 s, repeat for 40 cycles, 72°C 5 min, 10°C holding.

### 2.14 Visualization of gene expression profiling across developmental stages and species

Gene expression profiles of *PKHD1*/*Pkhd1*, *CYS1*/*Cys1*, *NDN*/*Ndn*, and *MYC*/*Myc* across kidney developmental stages in humans and mice, were downloaded from Evo-devo mammalian organs portal (https://apps.kaessmannlab.org/evodevoapp/) (Cardoso-Moreira et al., 2019). RPKM (reads per kilo base of transcript per million mapped reads) values were normalized using the highest value as 1 and the lowest value as 0 for each gene.

### 2.15 Statistical analysis

**FIGURE 1:**
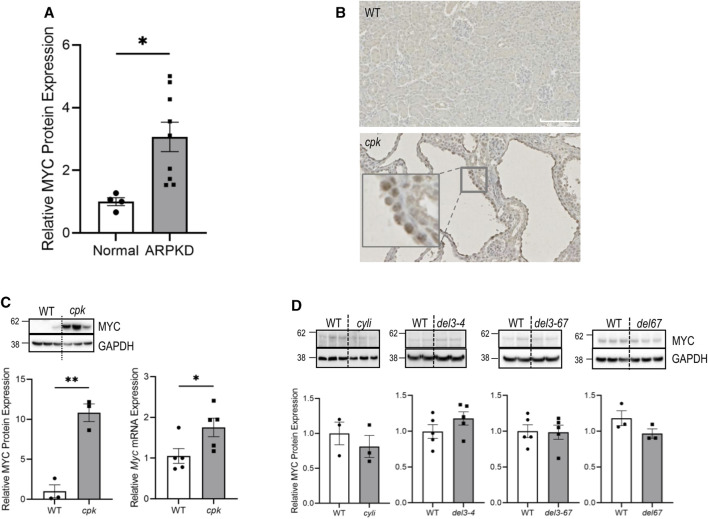
MYC/Myc overexpression is associated with renal cystic disease in human and mouse ARPKD. **(A)** Kidney lysates from normal human and ARPKD patients (Supplementary Table S1) were probed with anti-MYC and control anti-GAPDH antibodies. Relative MYC expression was normalized to GAPDH. Experiments were repeated twice independently. *t*-test *p < 0.05. Error bar indicates S.E.M. **(B)** Immunohistochemistry showing MYC expression (brown) in 14-day-old renal epithelial cells from wild-type (WT) and *cpk* mice. **(C)** Kidney lysates from 14-day-old WT and *cpk* mice were probed with anti-MYC and control anti-GAPDH antibodies. GAPDH was used for normalization (*cpk* mice, n = 6, *t*-test **p < 0.01). In parallel, *Myc* mRNA expression was quantified by qRT-PCR. The error bars indicate S.E.M. *indicates *p* < 0.05 **(D)** Kidney lysates from 14-day-old *Pkhd1* mutant mice (except 1 month old for *cyli*) and WT littermates were probed with anti-MYC and control anti-GAPDH antibodies, respectively. No significant differences were observed between groups. *cyli* (n = 3), *del3-4* (n = 5), *del3-67* (n = 5) and *del67* (n = 3). The error bars indicate S.E.M.

Non-parametric Wilcoxon sign rank test was used for analysis of qRT-PCR data normalized to control samples (Figure 1C, and 2D). All other data were analyzed using either two-way Student’s t-test or nonparametric test with GraphPad Prism version 9.1.2 for Windows, GraphPad Software, San Diego, California United States, www.graphpad.com.

## 4 Results

### 4.1 Elevated MYC expression in the kidneys of patients with ARPKD and in *cpk* mice with ARPKD-like kidney phenotype

MYC overexpression is a signature feature of cystic renal epithelia in human ADPKD and various mouse PKD models (Trudel, 2015). However, MYC expression in ARPKD has not been reported. In the current study, we analyzed MYC expression in ARPKD kidneys by immunoblotting. While MYC expression was marginally detectable in adult kidneys and a kidney from an infant without kidney disease (Supplementary Figure S4), we observed higher MYC abundance in all kidneys from patients with defined pathogenic variants in *PKHD1* (Figure 1A), with the highest MYC levels detected in kidneys from patients with *PKHD1* truncating pathogenic variants (Supplementary Table S1), resulting in the loss of FPC-CTD (AR1, AR2, AR3, and AR8) (Figure 1A and Supplementary Figure S4).

We then evaluated MYC expression in mouse models of ARPKD. Using IHC, we confirmed increased nuclear expression of mouse MYC protein in dilated collecting ducts from *cpk* kidneys (Figure 1B). Quantitative analysis confirmed 11-fold higher levels of MYC protein and 1.8-fold higher levels of *Myc* mRNA in the kidneys from *cpk* mice compared to WT mice (Figure 1C). In contrast, MYC protein levels were not elevated in kidneys from four different *Pkhd1* mutant mouse model lines (*Pkhd1* ^*cyli*^, *Pkhd1* ^*del3-4*^, *Pkhd1* ^*del3-67*^, or *Pkhd1* ^*del67*^) that did not exhibit a cystic kidney phenotype (Figure 1D). These data indicate an association between high MYC expression and the renal cystic phenotype in both human ARPKD and mice with an ARPKD-like kidney phenotype. Furthermore, the lack of enhanced MYC expression in *Pkhd1* mutant mice without cystic kidney phenotype suggests differences in the function of mouse and human FPC-CTDs.

### 4.2 Testing the phylogenetic conservation of extracellular and intracellular FPC domains in vertebrates

We note that in each of our mouse mutant lines, the predicted *Pkhd1* translated products would be missing the FPC-CTD. Furthermore, while the mouse and human FPC sequences are 73% identical overall, the CTDs share only 55% identity (Nagasawa et al., 2002). Therefore, we analyzed phylogenetic conservation of FPC in vertebrates to better understand potential differences in the regulation of MYC expression in renal epithelia derived from patients with ARPKD and *cpk* and *Pkhd1* mutant mice. We hypothesized that the low sequence conservation of the FPC-CTDs may contribute to functional differences among FPC orthologs; an important consideration given that mouse FPC undergoes Notch-like processing that releases the FPC-CTD, which can translocate to the nucleus.

To understand phylogenetic changes in *Pkhd1,* we queried the NCBI protein database for *PKHD1* orthologs and collected FPC protein sequences of 66 mammalian, 27 bird, 5 reptile, 3 amphibian, and 1 fish (Supplementary Table S4). The large variance in the number of FPC proteins in each class may in part reflect the number of sequenced vertebrate genomes in the NCBI data base. However, while genomic data are available for multiple fish, a *PKHD1* ortholog was identified only in the genome of *Latimeria chalumnae* (a coelacanth).

After alignment of 102 vertebrate FPC protein sequences, we used the WebLogo 3 entropy scores to evaluate FPC conservation across species (Supplementary Figure S1 and Supplementary Table S5 **)**. First, we compared the WebLogo 3 entropy scores for each of the conserved domains in the extracellular portion of FPC: five IPT domains, three TIG domains, two G8 domains, two PbH1 domains, and the TM domain with entropy scores from three regions of FPC that do not correspond to any conserved domains (Supplementary Figure S5B). Since the average WebLogo 3 entropy scores were similar (Supplementary Figure S5B), we then compared the WebLogo 3 entropy scores of FPC extracellular and cytoplasmic domains. Consistent with different protein sequence identities between human and mouse extracellular and cytoplasmic portions of FPC (Nagasawa et al., 2002) the WebLogo 3 entropy scores of vertebrate FPC extracellular domain were higher than entropy scores of FPC-cytoplasmic domains (Supplementary Figure S5C). This analysis supports the hypothesis that lower sequence conservation of mouse and human FPC cytoplasmic domain compared to extracellular domain may be functionally significant and potentially contribute to the phenotypic variability observed between *PKHD1* vs *Pkhd1* mutants.

### 4.3 Subcellular localization of mouse and human FPC-CTD and their effects on MYC expression

**FIGURE 2:**
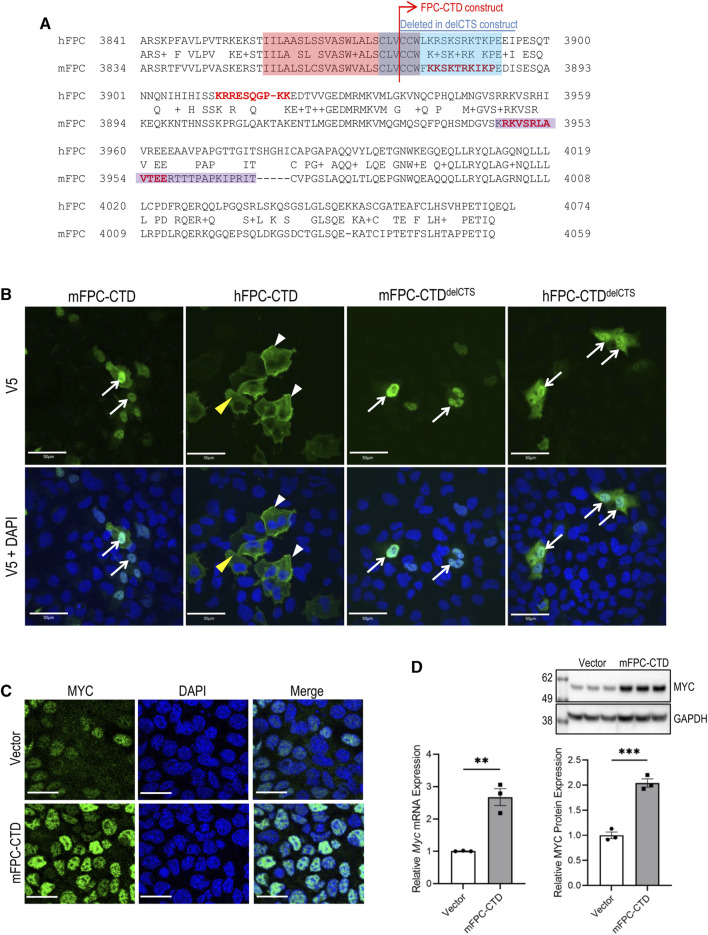
Subcellular localization of mFPC-CTD and hFPC-CTD and regulation of MYC expression by mFPC-CTD. **(A)** The alignments of human and mouse FPC-CTD. The red highlight indicates TM and blue highlight indicates the CTS. Predicted nuclear localization signals (NLSs) are shown in the red bold typeface. The purple highlight indicates experimentally tested NLS (Hiesberger et al., 2006). Blue line above the alignment indicates amino acids deleted from FPC-CTD to generate the FPC-CTD^delCTS^ constructs. **(B)** Transient transfection of V5-tagged mFPC-CTD, mFPC-CTD^delCTS^, hFPC-CTD, and hFPC-CTD^delCTS^, in mTERT-CCD or hTERT-HRE cells. Immunofluorescent staining was performed using anti-V5 antibody. White arrows—nucleus; yellow arrowheads—cytosol; white arrowheads—cell membrane; scale bars = 50 µM. **(C)** Immunofluorescent staining showing increased MYC expression in mIMCD-3 cells stably expressing mFPC-CTD. Green—MYC; blue—DAPI; scale bars = 20 µM. **(D)** Mouse *Myc* mRNA and MYC protein levels were increased in mIMCD-3 cells stably expressing mFPC-CTD. *Myc* mRNA expression was quantified by qRT-PCR. MYC protein expression was analysed with anti-MYC and normalized to GAPDH expression (N = 2, n = 3, *t*-test **p < 0.01, ***p < 0.001). Error bar indicates S.E.M.

Both human and mouse, FPC-CTD are encoded by *PKHD1*/*Pkhd1* exons 65, 66 and 67 (Supplementary Figure S5D). Overall, human and mouse FPC-CTD share 55% sequence identity, (Nagasawa et al., 2002). However, the CTS, localized in the human and mouse FPC-CTDs, are highly conserved (Figure 2A, red and blue highlights). Prior studies have experimentally validated one NLS in the mouse FPC-CTD (Figure 2A, purple highlight) (Hiesberger et al., 2006). However, using the web-based NLS prediction tool, SeqNLS (Lin and Hu, 2013), we identified two NLSs in mouse FPC-CTD (score >0.86), one of which overlapped with the experimentally identified NLS (Figure 2A, red bold text for predicted and purple highlight for experimentally validated NLS) (Hiesberger et al., 2006). On the other hand, human FPC-CTD had only one predicted NLS (score >0.86) (Figure 2A, red bold text). Interestingly, the two mouse, and the one predicted human NLS showed low sequence similarity.

While it has been determined that the mouse FPC-CTD translocates to the nucleus (Hiesberger et al., 2006), the intracellular trafficking of human FPC-CTD has not been determined and the nuclear function of FPC-CTD is not fully understood. Therefore, we compared intracellular localization of human and mouse FPC-CTD and tested their functions in the nucleus, concentrating on *MYC*/*Myc* regulation. Sequences the human and mouse FPC-CTD included the V5 tag and both the mouse and human FPC-CTDs contained the intracellular portion of the CTS (Figure 2A, blue highlight). The NLS sequences are shown in Figure 2A (red bold text).

By immunofluorescence, the mFPC-CTD localized primarily to the nucleus and was essentially absent from the cytoplasm (Figure 2B, left column). In contrast, the hFPC-CTD was largely excluded from the nucleus, localized to the cytoplasm and decorated the cell membrane (Figure 2B, second column from left). We suspected that nuclear trafficking of the hFPC-CTD construct with only one NLS was confounded by the CTS. Therefore, we deleted the CTS from both human and mouse constructs to generate plasmids expressing hFPC-CTD^delCTS^ and mFPC-CTD^delCTS^ respectively. Overexpression of these proteins showed strong nuclear localization for both human and mouse FPC-CTDs, although a fraction of hFPC-CTD^delCTS^ was retained in the cytoplasm (Figure 2B, right two columns).

We then investigated whether mFPC-CTD can regulate *Myc* expression in mIMCD-3 cells stably expressing the intact mFPC-CTD mouse construct. mIMCD-3 cells stably expressing the empty vector (pcDNA5) served as a control. This experimental approach allowed evaluation of the effect of mFPC-CTD on *Myc* expression in non-proliferating cells, 5 days post-confluence. The intensity of MYC immunostaining was higher in mIMCD-3 cells expressing mFPC-CTD than in the control cells (Figure 2C). Both *Myc* mRNA and MYC protein levels were higher in mIMCD-3 cells stably expressing mFPC-CTD, compared to control cells (Figure 2D). Taken together, our data provide the first evidence that overexpression of mFPC-CTD enhances MYC expression in cultured renal epithelial cells.

Efforts to perform these experiments with hFPC were confounded by the inability to generate an hTERT-HRE cell line that stably overexpressed either hFPC-CTD or hFPC-CTD^delCTS^. We observed that expression of hFPC-CTD was silenced after several passages of hTERT-HRE cells under selection, suggesting that stably expressed hFPC-CTD may be cytotoxic.

### 4.4 Mouse and human FPC-CTD bind to the *MYC/Myc* promoter and increase MYC/Myc expression

**FIGURE 3:**
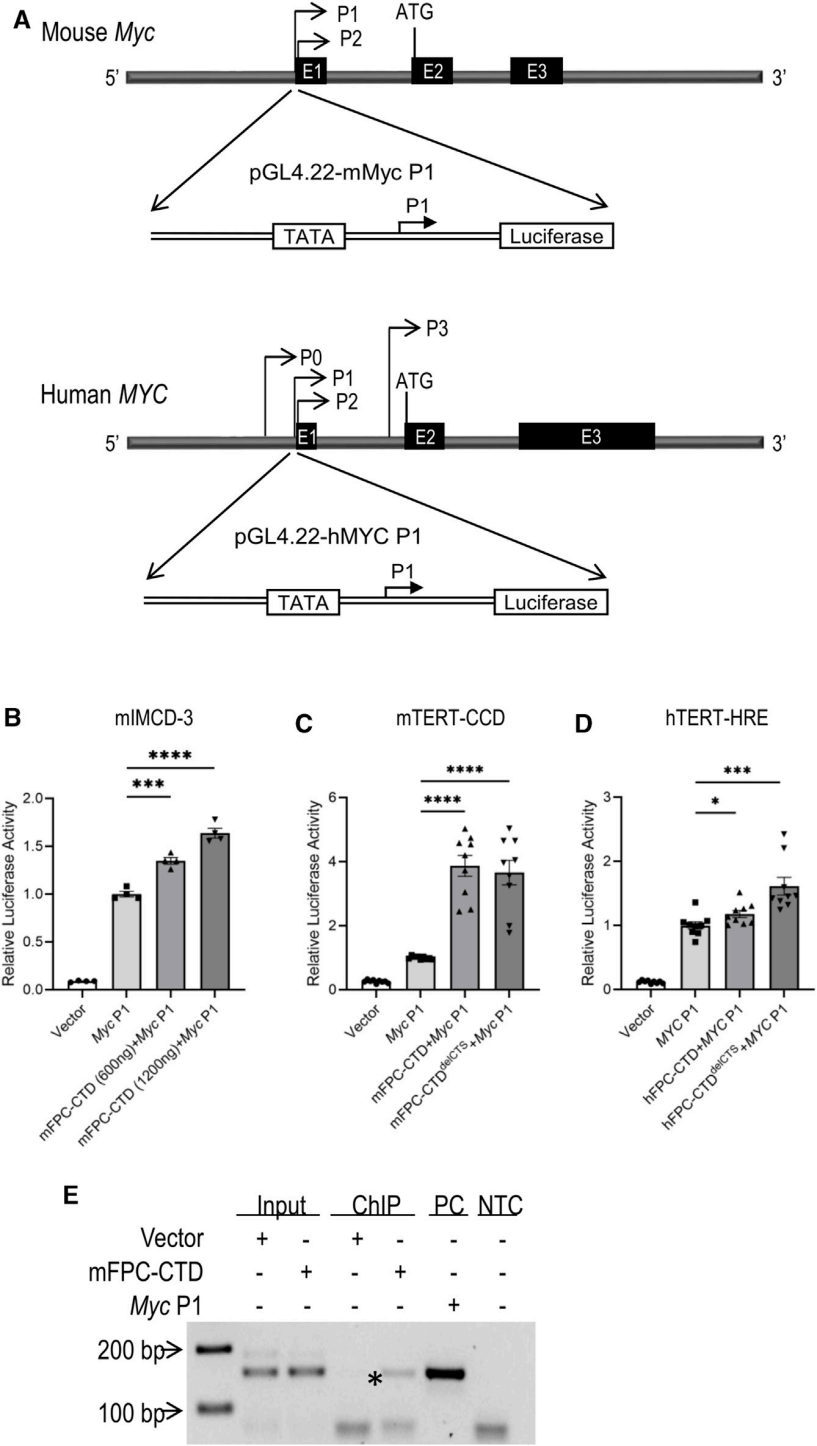
Activation of the *Myc*/*MYC* P1 promoter by m/hFPC-CTDs and binding of mFPC-CTD to the *Myc* P1 promoter **(A)** Schema of mouse (upper) and human (lower) *Myc*/*MYC* P1 luciferase reporter assay constructs. **(B)** The overexpression of mFPC-CTD increased *Myc* P1 promoter activity in mIMCD-3 cells in a dose-dependent manner. **(C)** Overexpression of mFPC-CTD and mFPC-CTD^delCTS^ increased *Myc* P1 promoter activity in mTERT-CCD cells. **(D)** hFPC-CTD^delCTS^ increased the *MYC* P1 promoter activity ∼1.6 fold in hTERT-HRE cells while hFPC-CTD only increased activity ∼1.1 fold. Experiments were repeated 3 times independently (N = 3) with 3 technical replicates (n = 3). The data were statistically analysed by combining all technical replicates (total n = 9) and *p < 0.05, ***p < 0.001, ****p < 0.0001. Error bar indicates S.E.M. **(E)** ChIP assay showing FPC-CTD binding to the endogenous *Myc* P1 promoter in mIMCD-3 cells. Asterisk indicates mFPC-CTD immunoprecipitation with endogenous *Myc* P1 promoter. PC indicates PCR amplification product using *Myc* P1 plasmid DNA as a template. NTC indicates negative, no template control. Experiments were repeated independently twice.

The mechanisms that govern *Myc* transcription are complex and involve multiple promoters (P0, P1, P2, and P3) and transcription start sites (Battey et al., 1983; Bentley and Groudine, 1986; Ray et al., 1987). The P1 and P2 promoters are the predominant *Myc* regulatory elements (Albert et al., 2001). In disease states, *Myc* overexpression primarily is driven from the P1 promoter (Wierstra and Alves, 2008). Therefore, we cloned the mouse *Myc* P1 and human *MYC* P1 promoter into a pGL4.22 reporter gene construct (Figure 3A). Co-transfection of mIMCD-3 cells with constructs expressing mFPC-CTD and *Myc* P1 promoter driven reporter gene showed dose-dependent activation of the *Myc* P1 promoter by mFPC-CTD (Figure 3B). *Myc* P1 promoter activation in mTERT-CCD cells was not affected by deletion of the CTS (Figure 3C). In comparison, co-transfection of hTERT-HRE cells with constructs expressing hFPC-CTD and *MYC* P1 reporter plasmid showed only minimal, though statistically significant activation of the *MYC* P1 promoter (Figure 3D). However, deletion of the CTS from the hFPC-CTD, which enhanced its nuclear localization, resulted in a 1.6-fold activation of the *MYC* P1 promoter (Figure 3D). These data demonstrate that both human and mouse FPC-CTDs can activate the *Myc*/*MYC* P1 promoter in cultured cells. Furthermore, we tested hFPC-CTD activation of *MYC* P1 promoter in mIMCD-3 cell line and found comparable activation in this mouse line as in the human cell line, hTERT-HRE (Supplementary Figure S6). However, we note that hFPC-CTD nuclear trafficking is regulated by the CTS.

To confirm the binding of mFPC-CTD to the *Myc* P1 promoter, we performed ChIP assays. We found that mFPC-CTD-V5 bound to the endogenous *Myc* P1 promoter in non-proliferating cells (Figure 3E, asterisk). As noted above, corresponding ChIP experiments with hFPC-CTD were confounded by our inability to generate an appropriate hTERT-HRE cell line.

### 4.5 Temporal expression of *PKHD1*/*Pkhd1*, *CYS1*/*Cys1*, *NDN*/*Ndn* and *MYC*/*Myc* mRNAs during pre- and post-natal kidney development in human and mouse

**FIGURE 4:**
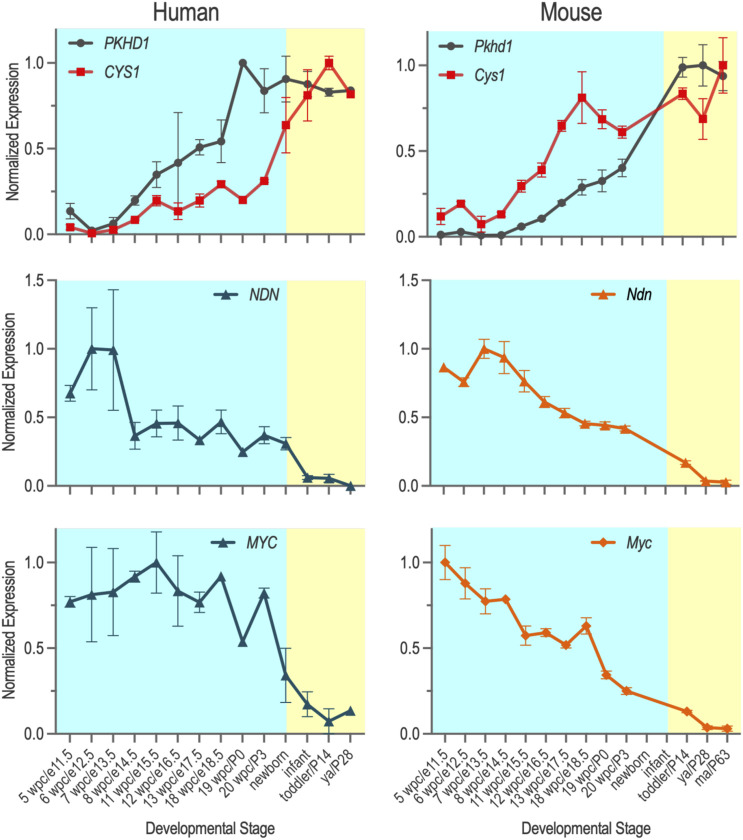
Temporal expression of *PKHD1/Pkhd1*, *CYS1/Cys1*, *CYS1/Cys1* and *MYC/Myc* mRNAs during pre- and post-natal kidney development in human and mouse were obtained from Evo-devo mammalian organs (https://apps.kaessmannlab.org/evodevoapp/), normalized, and graphed using GraphPad Prism. Y-axis shows normalized gene expression levels. X-axis shows kidney developmental stages that correspond to each other in humans and mice (human/mouse).

Considering the observed differences in the nuclear trafficking and function of human and mouse FPC-CTDs, we sought to better understand how species-specific regulation of *MYC/Myc* expression may contribute to the divergent renal phenotypes in human ARPKD and the *Pkhd1* mouse models. Therefore, we analyzed cystogene expression patterns in human and mouse kidneys during intrauterine and postnatal development using the Evo-devo mammalian organs portal (http://evodevoapp.kaessmannlab.org) (Cardoso-Moreira et al., 2019). We specifically focused on genes that are known to be mutated in human ARPKD (*PKHD1/Pkhd1*), in *cpk* mice (*CYS1/Cys1*), as well as necdin (*NDN/Ndn*), which we have previously shown regulates *Myc* expression (Wu et al., 2013). We normalized the kidney developmental stages of mouse and human during pre- and postnatal development. RPKM of *PKHD1*/*Pkhd1*, *CYS1*/*Cys1*, *NDN*/*Ndn*, and *MYC*/*Myc* genes were graphed at the corresponding developmental stages of human and mouse kidneys (Figure 4). These analyses demonstrated different timing of *PKHD1*/*Pkhd1* and *CYS1*/*Cys1* mRNA expression peaks during human and mouse kidney development (Figure 4, top panels). In the fetal human kidney, *PKHD1* mRNA levels progressively increase and reach maximal expression prior to birth, whereas the progressive increase in *CYS1* mRNA lags, reaching maximum expression in the post-natal period. Conversely, in the mouse kidney, maximum expression of *Cys1* mRNA precedes the peak of *Pkhd1* mRNA expression. Expression patterns of *NDN*/*Ndn* mRNA during human and mouse kidney development were similar (Figure 4, middle panels), with expression peaking in early developmental stages (6–8 weeks post conception and e12.5–14.5 in human and mouse kidney, respectively) and decreasing thereafter. Expression of *MYC*/*Myc* mRNA also peaked during early nephrogenesis and gradually decreased thereafter in both human and mouse kidneys (Figure 4, bottom panels).

With the assumption that mRNA expression serves as an appropriate proxy for protein levels, the species-specific differences in the *PKHD1*/*Pkhd1* and *CYS1*/*Cys1* developmental expression patterns and the observation that cystin may be protective of *MYC/Myc* activation suggest a mouse-specific renoprotective mechanism in mice lacking functional FPC. Therefore, we hypothesized that limiting cystin protein in kidneys from *Pkhd1* mutant mice may evoke dilatation of renal tubules and/or collecting ducts. To address this hypothesis, we crossed the *cpk* allele into mice that are homozygous for the *Pkhd1* ^*cyli*^ mutation (*cyli*), an indel in *Pkhd1* exon 48 that causes premature termination of protein translation (Yang et al., 2023). Kidneys from 10- and 12- month-old *cyli/cyli*;*cpk/+* mice on a mixed genetic background (D.B/11Ei; C57BL/6J, Supplementary Table S2) had mild tubular dilations that were absent in kidneys from age-matched *cyli/cyli*;+/+ mice (Supplementary Figure S7).

## 5 Discussion

Previous studies have demonstrated an association between MYC expression and renal cyst development in both human ADPKD and mouse PKD models (Trudel, 2015). MYC is overexpressed in cystic renal epithelial cells derived from ADPKD kidneys (Lanoix et al., 1996). Gene expression profiling studies demonstrated that the genes and pathways regulated by MYC are upregulated in kidneys from ADPKD patients (Husson et al., 2004; Song et al., 2009). Similar observations have been reported in mouse models of PKD (Trudel et al., 1998; Burtey et al., 2008; Kurbegovic and Trudel, 2013). Causality between MYC overexpression and renal cystogenesis is further suggested by observations in the SBM mouse model, in which a *Myc* transgene is driven by a β-globin promoter and SV40 enhancer (Trudel et al., 1991). Cystic kidney disease developed in transgenic mice overexpressing MYC, whereas mice that spontaneously lost the transgene did not develop renal cysts (Trudel et al., 1994). Additionally, *cpk* mice treated with *Myc*-antisense oligo exhibited reduced MYC protein expression, fewer renal cysts and improved renal function (Ricker et al., 2002).

In this study, we provide the first evidence that MYC is overexpressed in kidneys from patients with *PKHD1*-related ARPKD and confirm previous observations that MYC is upregulated in cystic renal epithelial cells from *cpk* kidneys (Ricker et al., 2002). In contrast, renal MYC expression levels were not altered in any of the *Pkhd1* mutant mice that lack a significant renal cystic phenotype. Our findings extend the proposition that MYC upregulation is a driver of the renal cystogenesis (Kurbegovic and Trudel, 2020).

Loss of functional FPC has different phenotypic consequences in human and mouse kidneys. In patients with pathogenic *PKHD1* sequence variants, even partial loss of FPC function can result in dramatic renal cystic disease (Cordido et al., 2021), suggesting that human FPC functions to maintain tubular integrity. In the absence of FPC, expression of MYC is aberrantly high leading to renal epithelial cell proliferation and cystogenesis. On the other hand, in mice lacking functional FPC, we show that *Myc* expression is not elevated and renal cysts are absent. A simple explanation could be that human *PKHD1* gene is important for kidney development and *MYC* homeostasis, while mouse *Pkhd1* plays a minimal role in nephrogenesis and *Myc* transcriptional regulation. This thesis is supported by a novel mouse line that was engineered to delete exon 67, which encodes most of the C-terminus, including the nuclear localization signal. Homozygous *Pkhd1* ^*del67*^ mice do not have a cystic phenotype (Outeda et al., 2017). In addition, a recent report describes a new model derived from the *Pkhd1* ^*del67*^ line such that exons 3-67 are deleted. Similar to homozygous *Pkhd1* ^*del67*^ mice, homozygous *Pkhd1* ^*del3-67*^ mutants do not express a renal cystic phenotype (Ishimoto et al., 2023). But is the explanation for the mouse-human phenotypic disparity so simple?

We and others have shown that mouse FPC-CTD is proteolytically cleaved through Notch-like processing (Kaimori et al., 2007; Follit et al., 2010; Cameron Varano et al., 2017). The FPC-CTD traffics into the nucleus and binds to double stranded DNA as a member of a ring-structure protein complex (Kaimori et al., 2007; Follit et al., 2010; Cameron Varano et al., 2017). However, it is not clear how FPC-CTD regulates gene expression, or which genes are regulated. To address this question and to explain the phenotypic disparity across species, we hypothesized that nuclear functions of hFPC-CTD and mFPC-CTD differ, particularly with respect to transcriptional regulation of *MYC/Myc* expression. As our experimental model, we employed *in vitro* overexpression to compare the nuclear trafficking and function of the human and mouse FPC-CTD.

Using immunofluorescence, reporter gene assays, and ChIP, we demonstrate that the mFPC-CTD traffics into the nucleus, binds to the *Myc* P1 promoter, and when overexpressed can activate *Myc* expression. In reporter gene assays, hFPC-CTD and mFPC-CTD have comparable functions; both activate *MYC/Myc* P1 promoter. However, we observed differences in cellular trafficking of these intracellular FPC fragments. While mFPC-CTD largely localized to the nucleus, hFPC-CTD remained associated with plasma membrane and localized in the cytoplasm, with translocation into nucleus only upon removal of CTS. This indicates that hFPC-CTD has the functional ability to activate *MYC* P1 promoter similar to mFPC-CTD, but the nuclear transport of these two proteins differs, suggesting that intracellular transport may in part explain the species-specific differences in function.

Our experimental findings regarding the FPC-CTD nuclear function raises a new conundrum about recessive PKD pathogenesis. We show that in ARPKD loss of function of human FPC-CTD (patients AR1, AR2, AR3, and AR8: Supplementary Table S1) is associated with aberrant overexpression of MYC in the cystic kidneys, suggesting that FPC functions as a negative regulator of *MYC* to prevent cystogenesis, as was previously shown for the pro-proliferative STAT3 (Dafinger et al., 2020). But our reporter assays indicate that hFPC-CTD upregulates *MYC*. It is important to note that this apparent paradox may reflect the difference between *in vivo* mechanisms where FPC-CTD-associated proteins may dictate specific regulatory function and reductionist reporter assays demonstrating the activation of a specific promoter by an overexpressed protein. Further studies will be necessary to decipher *MYC/Myc* transcriptional activation and inhibition during kidney development and how FPC-CTD, or the lack of it may contribute to *MYC/Myc* expression regulation.

While our studies provide novel information about the nuclear trafficking and function of human and mouse FPC-CTDs, a recent study identified mitochondrial targeting sequences in both mFPC-CTD and hFPC-CTD and demonstrated the trafficking of these proteins into the mitochondria (Walker et al., 2022). These developments imply that understanding both the nuclear and mitochondrial functions of human and mouse FPC-CTDs will be necessary to better define their roles in kidney cystogenesis.

Another key to human-mouse phenotype paradox may involve the *CYS1*/*Cys1* gene. In the mouse, cystin negatively regulates MYC expression through binding to mouse NDN and preventing its activation of *Myc* expression (Wu et al., 2013). While the mechanism of negative regulation is not completely understood, cystin could either compete with mouse NDN for binding to the *Myc* P1 promoter, or alternatively, cystin and mouse NDN could form a complex that binds to the *Myc* P1 promoter and inhibits its activity (Wu et al., 2013). Our data mining revealed that *Cys1* expression is upregulated before *Pkhd1* during mouse kidney development. In *cpk* mice, cystogenesis is initiated in the distal portion of developing proximal tubules at e16.5–17.5 and continues after birth (Preminger et al., 1982; Nidess et al., 1984; Avner et al., 1987). The activation of *Cys1* expression before *Pkhd1* in the developing mouse kidney and suppression of *Myc* expression by cystin could explain the absence of renal cystic phenotype in mice with mutant *Pkhd1*.

To better understand FPC function during development, we extended our study to analyze gene orthologs encoding FPC across phyla. These analyses suggest that the CTD may be evolutionary innovation associated with vertebrate transition from aquatic to terrestrial life. We found a *Pkhd1* orthologue in only one fish genome, the coelacanth *L. chalumnae*. This observation is consistent with a previous study that suggested the *Pkhd1* paralogue, *Pkhd1l1,* is the ancestral gene because *Pkhd1l1* gene is present in the Fugu (puffer fish) genome, but *Pkhd1* is not (Hogan et al., 2003). *PKHD1L1* and *PKHD1* are similar; both encode proteins that have a large extracellular segment with similar arrangement of conserved structural domains (Supplementary Figure S5A) and 41.5% protein sequence identity (Hogan et al., 2003). However, their cytoplasmic segments are quite different: human and mouse *PKHD1L1*-encoded proteins have very short cytoplasmic tails, eight and six amino acids, respectively, while hFPC-CTD and mFPC-CTD have 192 and 184 amino acids (Hogan et al., 2003).

In addition, our informatic analysis revealed higher sequence conservation of the FPC extracellular than intracellular domain across phyla, which may in part explain the difference in nuclear trafficking between mFPC-CTD and hFPC-CTD. The mFPC-CTD has two predicted canonical NLSs, one of which has been experimentally validated (Hiesberger et al., 2006). In contrast, hFPC-CTD has only one predicted NLS. The difference in the number of NLSs in human and mouse FPC-CTD could explain the differences in the distribution of hFPC-CTD and mFPC-CTD in the nucleus *versus* other subcellular compartments, e.g., mitochondria. This finding raises an intriguing possibility that will require further study.

Finally, we used data mining to compare the temporal expression of *PKHD1/Pkhd1* and *CYS1/Cys1* in developing human and mouse kidneys respectively. In human kidneys, maximal expression of *PKHD1* preceded *CYS1*. However, during mouse kidney development, *Cys1* expression is upregulated before *Pkhd1* (Figure 4). Assuming similar regulation of *MYC/Myc* gene expression by human and mouse cystin in association with NDN, our data suggest that 1) *MYC*/*Myc* expression can be activated by human and mouse NDN during early kidney development and factors that regulate *MYC*/*Myc* expression in later stages of kidney development differ in humans and mice; and 2) during middle to late stages of mouse nephrogenesis, *Myc* gene expression is downregulated by cystin in the mouse kidney but much less than in the human kidney. Therefore, the differences in the temporal expression pattens of *CYS1*/*Cys1* could contribute to the relative renoprotection from cystogenesis in *Pkhd1*-deficient mice.

In summary, we provide the first report of elevated MYC levels in *PKHD1*-deficient human kidneys. In contrast, we show that MYC abundance is unaltered in non-cystic kidneys from *Pkhd1*-deficient mice. We demonstrate several key differences between human and mouse that may explain the relative renoprotection in *Pkhd1*-deficient kidneys: 1) differences in the number of NLS in the FPC-CTD; 2) differential impact of the human and mouse CTS on intracellular trafficking and subcellular distribution of the FPC-CTD; and 3) differences in the temporal expression of *PKHD1/Pkhd1* and *CYS1/Cys1* during nephrogenesis. In addition, we observed that reduced cystin levels in *Pkhd1*-deficient mice lead to renal tubular dilatation, suggesting that in mice both cystin and FPC-CTD are necessary to maintain renal tubular architecture. Given the limited sequence identity of human vs mouse FPC-CTD, we speculate that the cytosolic cleavage peptides may have different protein interacting partner(s) and these protein complexes may differentially regulate *MYC/Myc* expression *in vivo*. Taken together, our data extend previous observations and indicate that MYC dysregulation is a central driver of renal cystogenesis in both ARPKD and ADPKD.

## Data Availability

The datasets presented in this study can be found in online repositories. The names of the repository/repositories and accession number(s) can be found in the article/Supplementary Material.
