# Supplementary figures and images for: Differential regulation of MYC expression by PKHD1/Pkhd1 in human and mouse kidneys: phenotypic implications for recessive polycystic kidney disease
Source: Front Cell Dev Biol. 2023 Nov 17;11:1270980. doi: 10.3389/fcell.2023.1270980 (PMC10731465; doi:10.3389/fcell.2023.1270980)

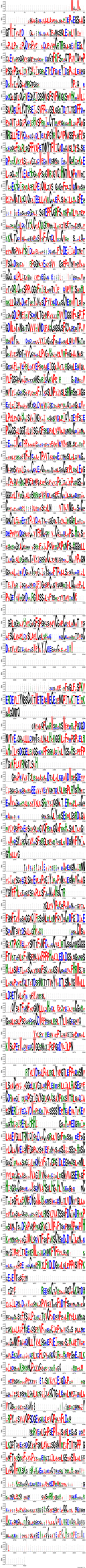

Supplement: Supplementary file 1 [file DataSheet2.pdf]
