# Supplementary material for: Differential regulation of MYC expression by PKHD1/Pkhd1 in human and mouse kidneys: phenotypic implications for recessive polycystic kidney disease
Source: Front Cell Dev Biol. 2023 Nov 17;11:1270980. doi: 10.3389/fcell.2023.1270980 (PMC10731465; doi:10.3389/fcell.2023.1270980)
Supplement: Supplementary file 3 [file DataSheet1.docx]

Supplementary Material

Differential Regulation of MYC Expression by *PKHD1/Pkhd1* in Human and Mouse Kidneys: Phenotypic Implications for Recessive Polycystic Kidney Disease

Naoe Harafuji^1†^, Chaozhe Yang^1†^, Maoqing Wu^1^, Girija Thiruvengadam^1^, Heather Gordish-Dressman^1^, R. Griffin Thompson^2^, P. Darwin Bell^2^, Avi Z. Rosenberg^3^, Claudia Dafinger^4^, Max C. Liebau^5^, Zsuzsa Bebok^2^, Ljubica Caldovic^6,7^ and Lisa M. Guay-Woodford^1,6*^

*** Correspondence:** Lisa M. Guay-Woodford, M.D.: [guaywoodfl@chop.edu](mailto:guaywoodfl@chop.edu)

# Supplementary Figures

Please see separate PDF document: Supplementary Figure S1_Weblogo3 (PDF)

**Supplementary Figure S1.** **The graphical representation of FPC amino acid sequence conservation** (PDF). 102 FPC protein sequences (**Supplementary Table S4**) were aligned using Clustal Omega and the alignment was visualized using WebLogo 3.

**
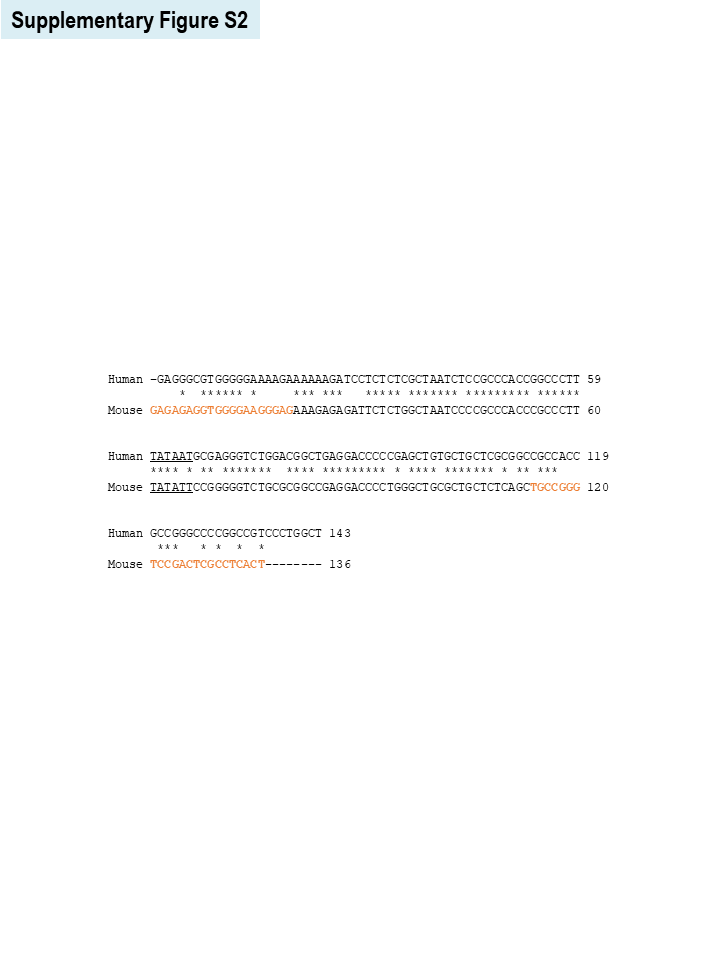
**

**Supplementary Figure** **S2.** **Sequence alignment of human and mouse *MYC*/*Myc* P1 promoter**. Human and mouse *MYC*/*Myc* P1 promoter sequences were aligned. Underline indicates the TAT box. Orange text indicats PCR primers that were used to amplify the full-length mouse *Myc* P1 promoter for ChIP assay (**Figure 3E**).


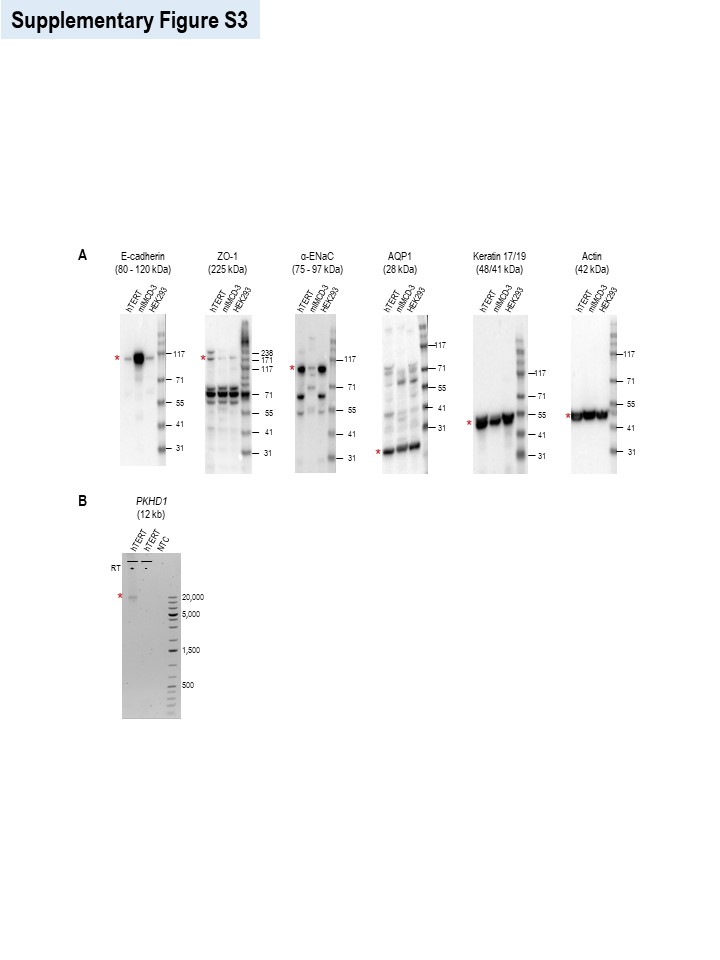


**Supplementary Figure** **S3.** **Renal epithelial character of human TERT line was analysed by western blotting (WB) and RT-PCR.** (A) Expression of renal epithelial cell markers in hTERT-HRE cells. HEK293 and mIMCD-3 were used as positive controls. E-cadherin was used as an epithelial marker. Expected size of glycosylated E-cadherin is ~120 kDa and unglycosylated is ~80 kDa. ZO-1 is a tight junction protein; expected size is 225 kDa. α-ENaC was used as a marker for renal epithelial cells. Expected size of glycosylated α-ENaC is ~97 kDa and unglycosylated is ~75 kDa. AQP1 was used as a marker for renal tubule cells, expected size is 28 kDa. Keratin 17/19 was used as an epithelial cell marker; expected size is 48/41 kDa. Actin was used as a loading control; expected size is 42 kDa. Red asterisks indicate expected protein sizes. (B) *PKHD1* mRNA expression in hTERT-HRE was tested by RT-PCR. Red asterisk indicates expected *PKHD1* mRNA sizes. With (+) and without (-) reverse transcriptase (RT). No template control (NTC).

**
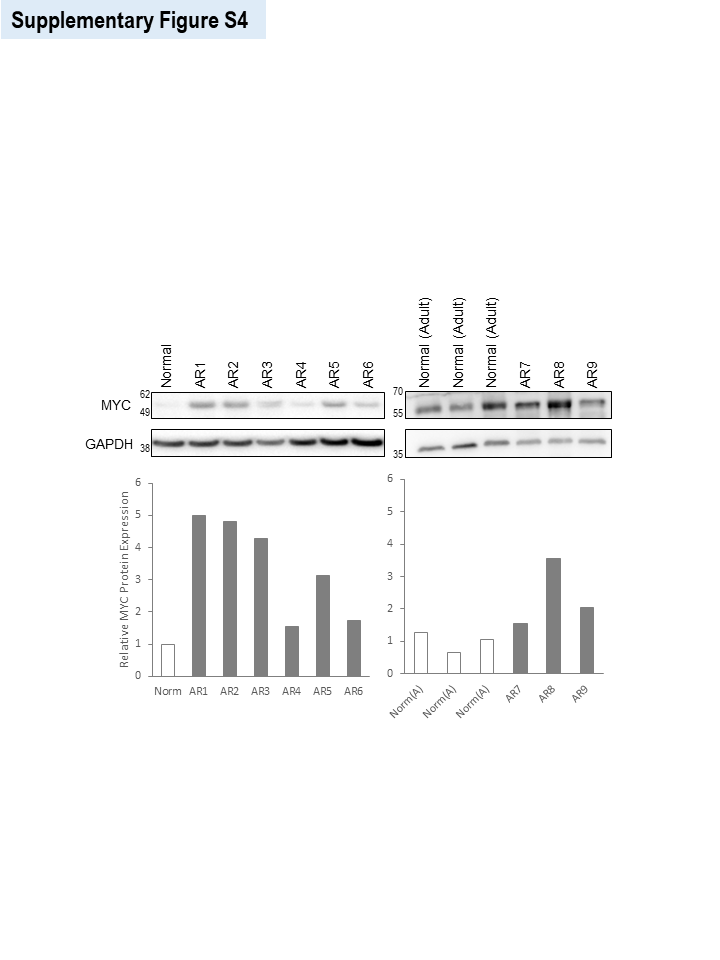
**

**Supplementary Figure S4.** **MYC upregulation is associated with renal cystic disease in human ARPKD.** Kidney lysates from individuals without renal cystic disease and patients with ARPKD (**Supplementary Table S1**) were probed with anti-MYC and control anti-GAPDH antibodies. Experiments were repeated twice independently.


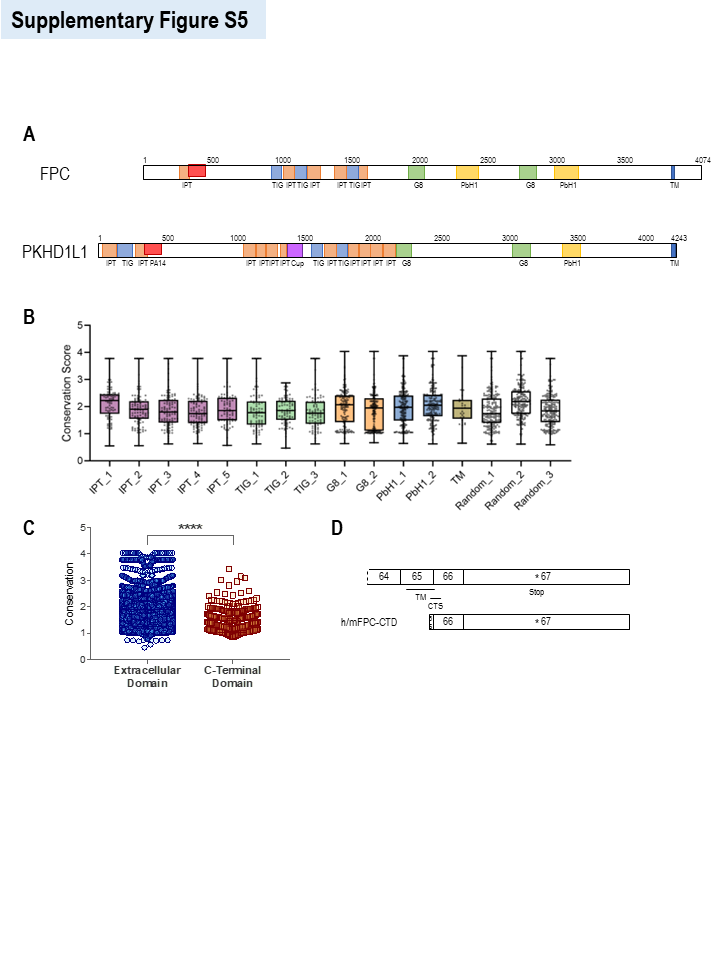


**Supplementary Figure S5.** **Phylogenetic conservation of FPC protein sequences.** (A) Schema of FPC and PKHD1L1 Proteins. Conserved domains listed by NCBI CDD were presented by boxes with colours: orange– IPT, red – PA14, blue – TIG, green – G8, yellow – PbH1, navy – TM, purple – Cupredoxin domain of PKHDL1. (B) WebLogo 3 was used to analyze conservation scores of individual amino acids in the alignment of 102 FPC protein sequences. Average of WebLogo 3 conservation scores for all domains were similar to randomly selected 3 regions of FPC that do not correspond to any conserved domains (Random 1-3). (C) Conservation scores of amino acids in the FPC extracellular domain and CTD. **** indicates p-value < 0.0001. (D) Diagrams of *PKHD1/Pkhd1* exons coding for the FPC TM domain and FPC-CTD. Both human and mouse FPC-CTD constructs contain the C-terminus of the transmembrane domain (TM) and most of the ciliary targeting sequence (CTS) from exon 65. The stop codons are located in the middle of exon 67.


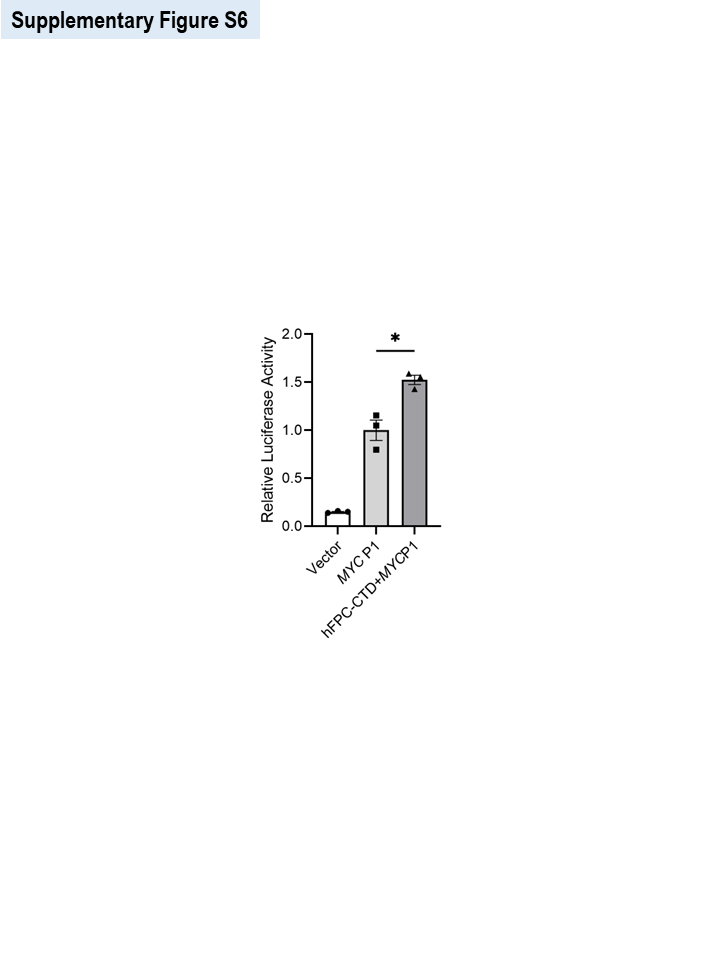


**Supplementary Figure S6. hFPC-CTD activates *MYC* P1 promoter in mIMCD-3 mouse cell line.** The constructs, expressing human FPC-CTD and human *MYC* P1 luciferase reporter gene, were co-transfected into the mIMCD-3 mouse cell line. Activation of the *MYC* P1 promoter by hFPC-CTD was similar in mIMCD-3 mouse cell line and human hTERT-HRE cell line. The bars indicate mean±S.E.M., n=3; * indicates p<0.05.


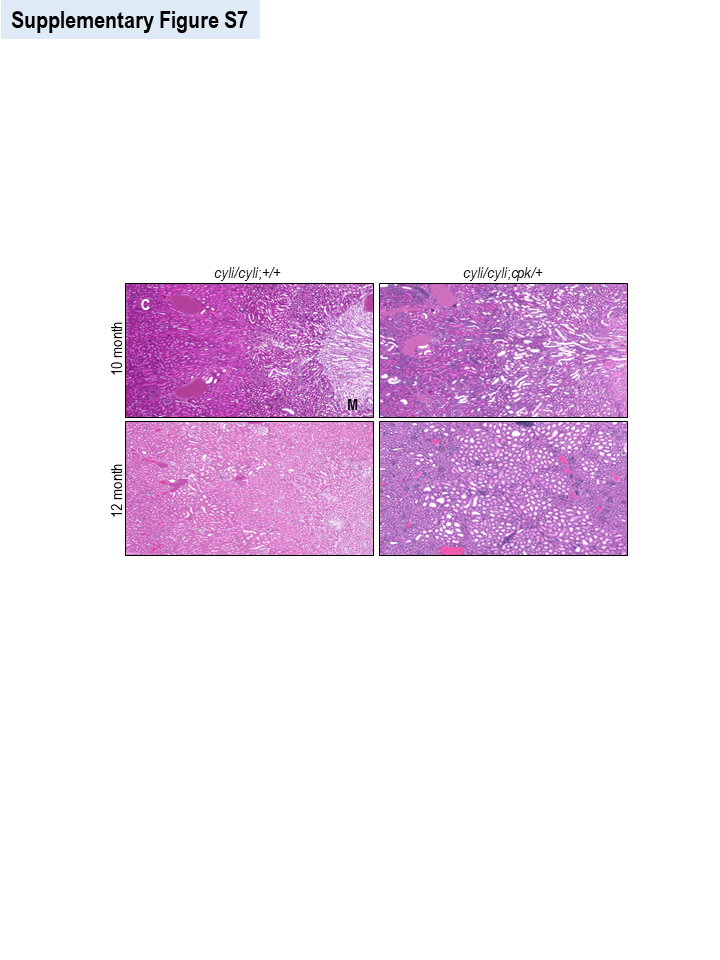


**Supplementary Figure S7. Haploinsufficiency of the cystic kidney phenotype in *cyli/cyli*;*cpk/+* mutant mice.** The *cyli/cyli Pkhd1* mutant mice were crossed with *cpk/+* mice to generated *cyli/cyli*; *cpk/+* and *cyli/cyli*; *+/+* littermate. The mice were aged for 10 and 12 months and their kidney phenotype was examined with hematoxylin and eosin staining. The histological analysis of kidneys from 10 and 12 months old *cyli*/*cyli*;*cpk*/+ mice, that have mixed genetic background (D.B/11Ei; C57BL/6J), revealed minor and patchy tubular dilations (**right panel**) that were absent in kidneys from the *cyli*/*cyli*;+/+ mice (**left panel**). C indicates cortex and M indicates medulla. We analyzed 1 pair of 10-month-old and 2 pairs of 12-month-old mouse kidneys.

# Supplementary Tables (EXCEL)

# Supplementary Materials and Methods

## RT-PCR

cDNA was synthesized as described in the Materials and Methods section. PCR was performed with primers specific for *Pkhd1* exons 1 (forward: 5’-CAT TTG AGG CAC AAG GCT GAC ACA-3’) and 67 (reverse: 5’-CTG AGG TCT GGG CGT AAC AG-3’) sequences. PCR amplicons were visualized by 0.7 % agarose gel.
