# Supplementary material for: Differential regulation of MYC expression by PKHD1/Pkhd1 in human and mouse kidneys: phenotypic implications for recessive polycystic kidney disease
Source: Front Cell Dev Biol. 2023 Nov 17;11:1270980. doi: 10.3389/fcell.2023.1270980 (PMC10731465; doi:10.3389/fcell.2023.1270980)

Figure 1A and Supplementary Figure S4 _raw images

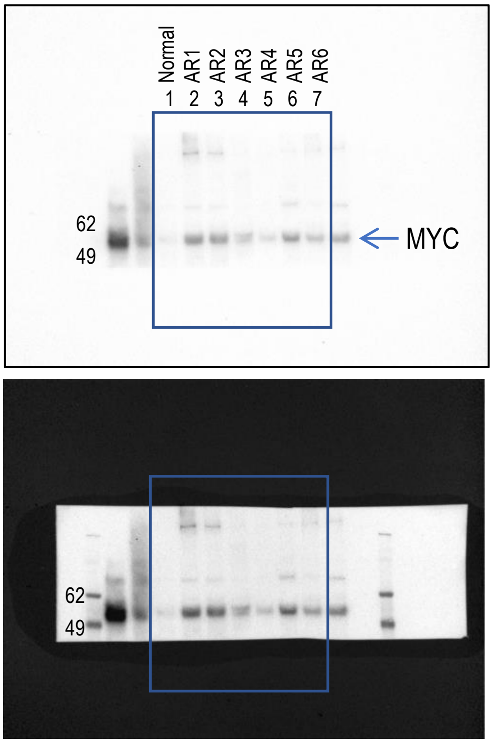

IB: MYC (Abcam # ab32072, 1:1K)

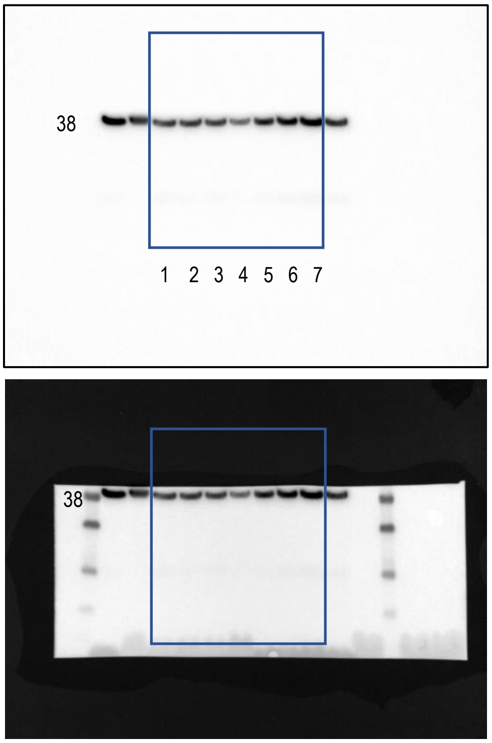

IB: GAPDH

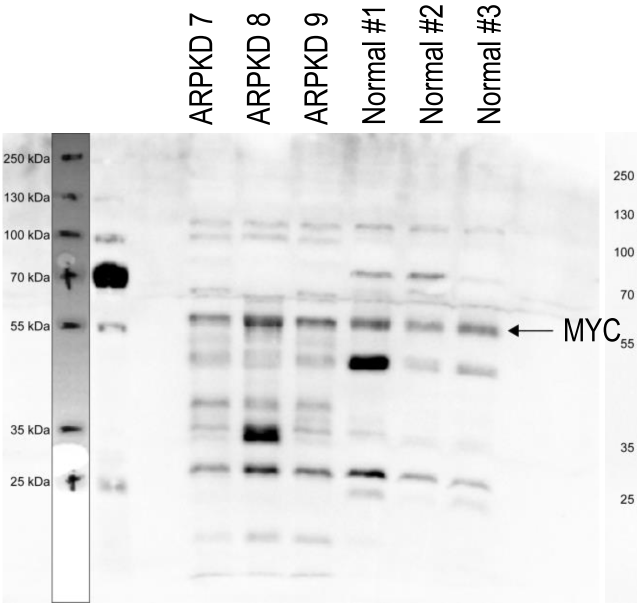

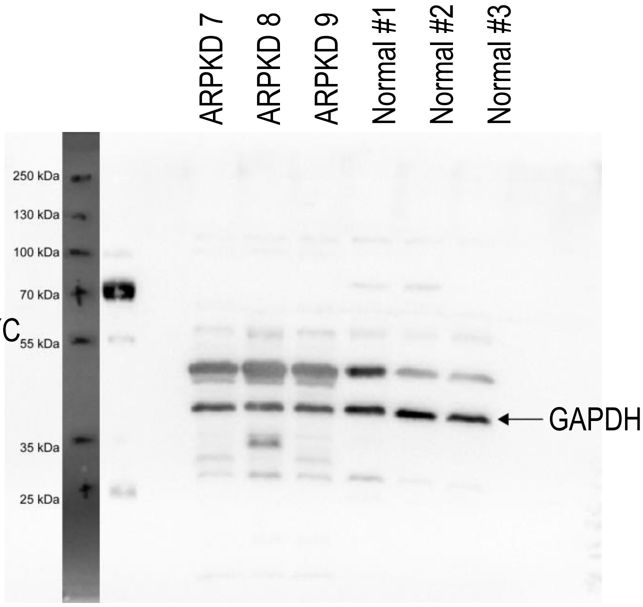

IB: MYC (Cell Signaling # 2276, 1:1K)

Figure 1C_raw images

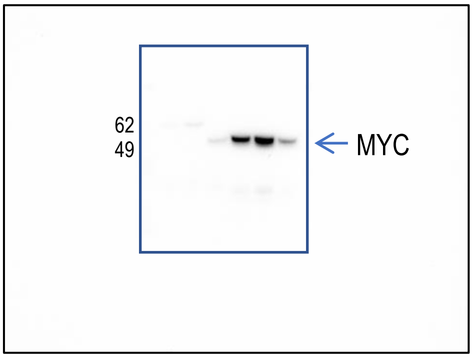

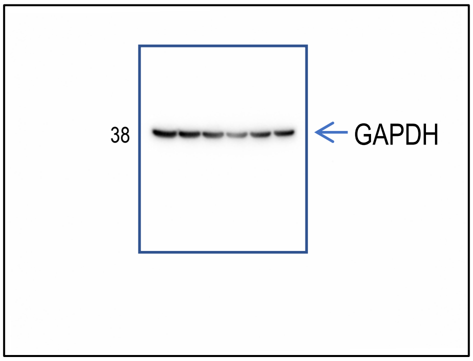

IB: GAPDH

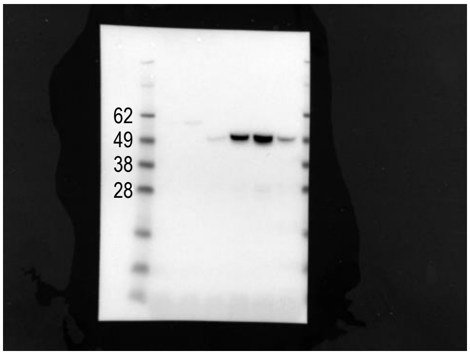

IB: MYC (Abcam# ab32072, 1:1K)

Figure 1D_raw images

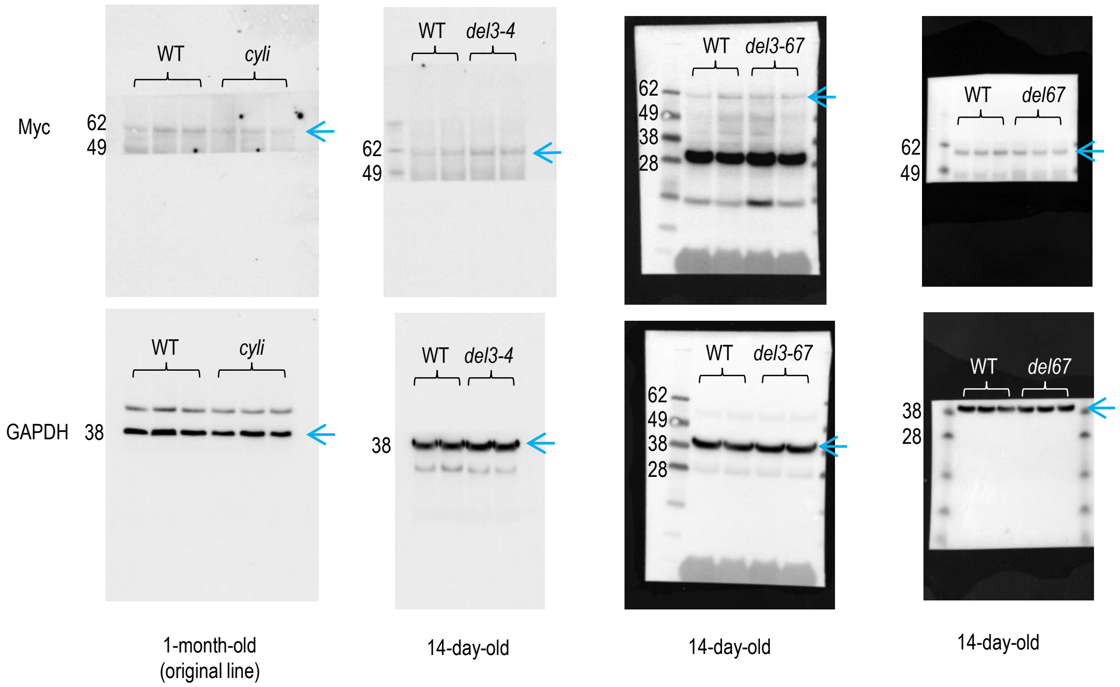

Figure 2D_raw images

62
49

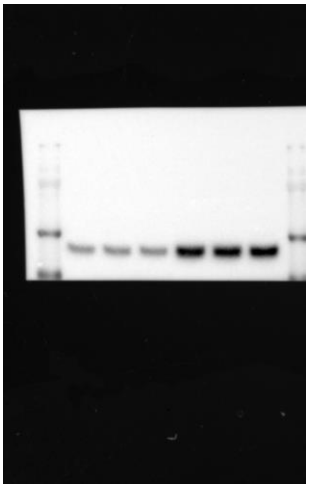

MYC

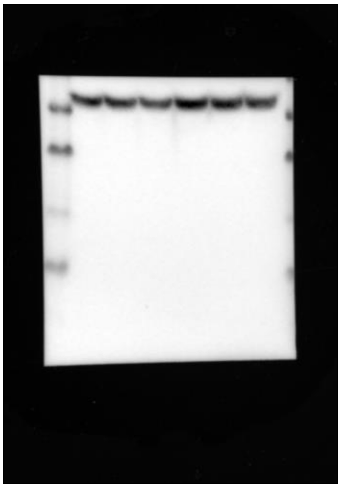

38
28

GAPDH

Figure 3E_raw images

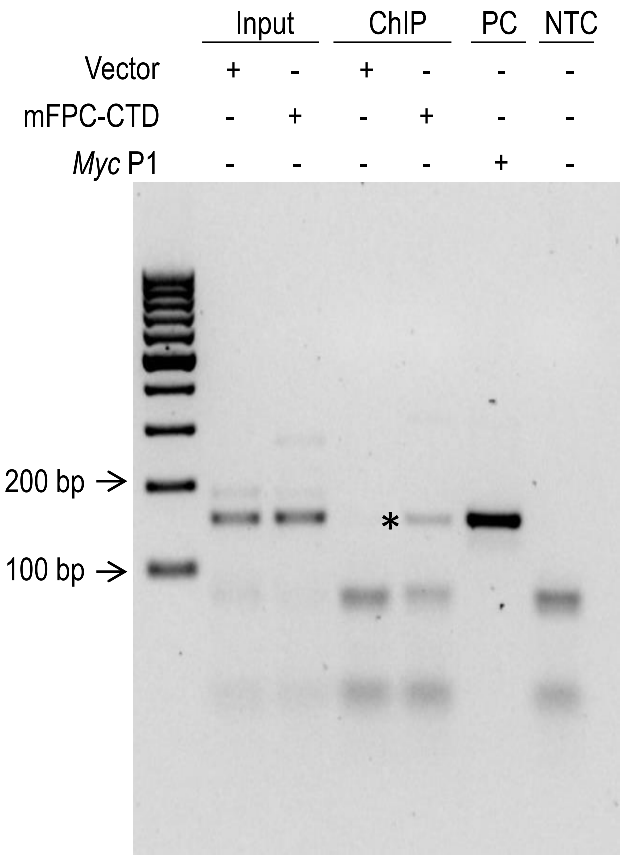

Supplement: Supplementary file 4 [file Image1.pdf]
